# Conservation of Energy: Missing Features in Its Nature and Justification and Why They Matter

**DOI:** 10.1007/s10699-020-09657-1

**Published:** 2020-04-04

**Authors:** J. Brian Pitts

**Affiliations:** 1 Faculty of Philosophy, University of Cambridge, Cambridge, UK; 2 School of History and Heritage, University of Lincoln, Lincoln, UK

**Keywords:** Conservation laws, Mental causation, Energy, Uniformity of nature, Locality, Symmetries

## Abstract

Misconceptions about energy conservation abound due to the gap between physics and secondary school chemistry. This paper surveys this difference and its relevance to the 1690s–2010s Leibnizian argument that mind-body interaction is impossible due to conservation laws. Justifications for energy conservation are partly empirical, such as Joule’s paddle wheel experiment, and partly theoretical, such as Lagrange’s statement in 1811 that energy is conserved if the potential energy does not depend on time. In 1918 Noether generalized results like Lagrange’s and proved a converse: symmetries imply conservation laws and vice versa. Conservation holds if and only if nature is uniform. The rise of field physics during the 1860s–1920s implied that energy is located in particular places and conservation is primordially local: energy cannot disappear in Cambridge and reappear in Lincoln instantaneously or later; neither can it simply disappear in Cambridge or simply appear in Lincoln. A global conservation law can be inferred in some circumstances. Einstein’s General Relativity, which stimulated Noether’s work, is another source of difficulty for conservation laws. As is too rarely realized, the theory admits conserved quantities due to symmetries of the Lagrangian, like other theories. Indeed General Relativity has *more* symmetries and hence (at least formally) *more* conserved energies. An argument akin to Leibniz’s finally gets some force. While the mathematics is too advanced for secondary school, the ideas that conservation is tied to uniformities of nature and that energy is in particular places, are accessible. Improved science teaching would serve the truth and enhance the social credibility of science.

## Introduction

The idea is that any causal interaction between mind and matter would violate the principle of the conservation of energy....So much the worse, it seems, for interactionism. (Though traditional, the argument is still current; for example, Dennett endorses it (1991, pp. 34–35)).

This argument is flawed....

In short, physicalists need to be wary of bad reasons to think physicalism is true, arising from naivety about physics. (Butterfield 1997, pp. 146, 147)

The conservation of energy is a topic that has frequently arisen over the centuries within the philosophy of mind, often as an objection to broadly Cartesian mental causation. This paper will briefly summarize the history of this objection (Pitts 2020b) before exploring features of conservation laws as presently understood by physicists but not widely known among philosophers. This argument, due to Leibniz in the seventeenth century and often repeated until today, begs the question (Pitts 2019; Cucu and Pitts 2019). That point has certainly been made before, both in modern times (Averill and Keating 1981) and in the eighteenth century, but not with full mathematical detail on conservation laws, it seems.

It has been noticed recently that General Relativity finally gives this kind of argument a measure of force (Pitts 2020a). How secondary school treatments of conservation laws might be improved without the need advanced mathematics or logic will then be explored, as will the reasons that these improvements are important.

## Mental Causation and/or Conservation Laws, 1690s–1760s

As early as the 1690s Leibniz publicly deployed his understanding of conservation laws for momentum (due to Wallis, Wren and Huygens Hugens 1669; Wallis 1668; Wren 1668) and his own proposed law of the conservation of living force (vis viva), an ancestor of energy, as reasons to reject Cartesian soul-to-body causation (Leibniz 1969, 1981, 1985, 1997; Pitts 2020b). In the *Theodicy* he puts the matter thus:

...two important truths ...have been discovered since M. Descartes’ day. The first is ...[conservation of $$ mv^2,$$ not |*v*|.] The second discovery ...[involves directionality: momentum $${\vec{p}}=m {\vec{v}}$$ is a vector.] If this rule had been known to M. Descartes,...I believe that that would have led direct [*sic*] to the Hypothesis of Pre-established Harmony, whither these same rules have led me. For apart from the fact that the physical influence of one of these substances on the other is inexplicable, I recognized that without a complete derangement of the laws of Nature the soul could not act physically upon the body. (Leibniz 1985, p. 156)

Leibniz was of course taking on board scientific progress in speaking of a conserved momentum vector rather than Descartes’s conservation of quantity of motion (volume $\cdot$ speed). Whether Leibniz’s conservation of vis viva, proportional to mass and the square of speed, was right was unclear till the nineteenth century.

It is unclear whether Descartes wanted the conservation of motion to hold even in the presence of mental causation and exploited his directionless conservation of motion to that end (as Leibniz claims). Apparently he never said this (Remnant 1979; Garber 1983). “The overwhelming impression that one gets from the texts is that Descartes just was not very concerned about reconciling his interactionism with his conservation law.” (Garber 1983) Indeed

there is reason to believe that Descartes may never have been committed to the position that his conservation law holds universally and may have allowed for the possibility that animate bodies lie outside the scope of the laws that govern inanimate nature. (Garber 1983)

In any case some Cartesians did hold this view (Woolhouse 1986; Schmaltz 2008, pp. 172, 173), making it a view worth consideration and critique.

Whereas in the last 170-odd years many have used Leibniz’s argument as a reason to deny the existence of souls altogether in favor of materialism, Leibniz took dualism for granted but denied the soul-body *interaction* posited by Descartes. One might almost say that soul-body interaction was also motivated by common sense. Everyone is familiar with eating more (say) pizza because one likes the taste; it seems that the pizza causes a taste experience enjoyed by the mind, which then causes bodily motions to put more pizza into the mouth, which gives pleasure to the mind, etc.: iterated body-to-soul and soul-to-body causation. Or consider more elementary experiences. If I decide to raise my arm, my arm goes up. If I stub my toe on a rock, then I feel pain. These seem to be examples of mental-to-physical causation and physical-to-mental causation, respectively. Leibniz’s own “machine” or “mill” argument seems to undermine physicalism (Rescher 1991, section 17): no understanding of how mechanical parts interact gives one the least idea of how “perception” occurs, as one realizes by imagining examining the workings of a scaled-up version of the machine. But Leibniz introduced the dualist *non*-interactionist idea of “pre-established harmony,” according to which there is mental-to-mental causation and physical-to-physical causation but no interaction between the two realms; there is only a divinely orchestrated harmony between the two.^1^ Thus if I stub my toe, the pain is not caused by my toe-stubbing, but by some (secret?) prior mental cause. If I decide to raise my arm and my arm goes up, the arm-rising is not caused by my decision to raise my arm, but by some (secret?) physical cause. Thus as far as causation in the created realm is concerned, our mental lives could happen in exactly the same way if there were no physical world, and the physical world could happen in exactly the same way if we had no mental lives. The former point was viewed by critics as an objection in that it made God’s creating the physical world pointless, far from having the sufficient reason that all of God’s actions were claimed by Leibniz to have. The latter point is more closely related to various contemporary philosophical debates.

How does Leibniz’s argument from causation serve some contemporary purposes? In the early-mid eighteenth century the three plausible views on the market were broadly Cartesian interactionism, Malebranche’s occasionalism, and Leibniz–Wolff pre-established harmony. Occasionalism held that there is no causal influence from finite minds (such as ours) to the physical world; only God, an infinite mind, could act on the physical world, because it would be impossible for God’s willings not to come true. In the nineteenth century the idea of epiphenomenalism arose due to Huxley: there is physical-to-mental causation but not mental-to-physical. Epiphenomenalism can be (but need not be) a form of substance dualism. Contemporary uses of Leibniz’s anti-interactionist argument usually takes for granted that interactionist dualism is the only philosophically plausible form of dualism. If interactionist dualists are not all that common today [though there seems to be a recent increase in respect for the view (Lycan 2009, 2013, 2018)], proponents of pre-established harmony, occasionalism, and substance dualist epiphenomenalism are far rarer, so this belief about the relatively greater plausibility of *interactionist* dualism seems uncontroversial today—in marked contract to France in 1700 or Germany in 1730. Thus Leibniz’s anti-interactionist argument serves as an ostensibly scientific subargument that refutes substance dualism by refuting interactionism, the most plausible version. His anti-interactionist argument, if successful, also refutes the possible view of interactionist *property* dualism, that there are mental properties (but no mental substance) and these properties can act^2^ on the physical world (Searle 2004, pp. 44–46; Zimmerman 2007; Crane 2001, pp. 40, 43, 50).

## Some Eighteenth Century Physicists’ Implicit Views: Newton and Euler

Leibniz’s argument faced serious opposition, whether tacit or explicit, in the eighteenth century; while Leibnizian pre-established harmony gained the upper hand in Germany for a while, eventually interactionism recovered as the dominant view even there (Watkins 1995a, b, 1998; Priestly 1777, p. 64). This opposition included not only some very good philosophers such as Crusius and Knutzen in Germany, but also, one can argue, at least implicitly the two best physicists of the eras of Leibniz and Wolff, namely, Newton and Euler (Pitts 2020b). It is difficult, perhaps impossible, to find either Newton or Euler explicitly addressing Leibniz’s argument, but it seems clear what they thought or should have thought given what they did say.

Newton’s unpublished work often showed his belief in mental force, as in a draft of *Opticks*:

Seeing therefore the variety of motion (wch we see) in the world is always decreasing, there is a necessity of conserving and recruiting it by active principles; such as are (the power of life and Will by which animals move their bodies with great and lasting force;) (McGuire 1968, pp. 169, 170, bracketed and cancelled)

Newton in fact quite *frequently* affirmed strong views of mental causation (Dempsey 2006). He seems not to have been worried by the idea of conservation law failure due to mental force.^3^ It is quite obvious that momentum conservation fails due to lack of the action-reaction otherwise guaranteed by Newton’s third law of motion. If the mind exerts some influence on the body, almost certainly the body does not exert an equal and opposite force on the mind—it is not at all clear what such a claim could even mean—so momentum is not conserved. While I am not aware of Newton’s explicitly addressing Leibniz’s argument, it is quite evident what he should have thought about it: one should simply accept non-conservation of momentum due to mental force. Non-conservation of energy (or rather its ancestor vis viva, $mv^2$) was already accepted by Newton and many others because of inelastic collisions, so momentum (non)conservation would be the only serious question for a Newtonian. Relatedly, in the eighteenth century the vis viva controversy raged over the proper measure of force: whereas the conservation of momentum and the conservation of energy now are seen as complementary, in that era it was thought that there should be a “true measure of force,” which was conserved in physical interactions (Clarke 1727; Hankins 1965; Laudan 1968; Iltis 1970, 1971; Gale 1973; Heimann 1977; Papineau 1977; Terrall 2004; Smith 2006; Rey 2018). The conservation of momentum was accepted on both sides—one notes that it follows immediately from Newton’s second and third laws of motion—but the conservation of vis viva was a controversial claim allied to claims that vis viva was the true measure of force.

Leibniz’s argument seems to have been equally unimpressive to Leonhard Euler. If Newton was the dominant physicist of the late seventeenth century, Euler was similarly dominant in the mid-eighteenth century. He was one of the greatest mathematicians of all time and the most prolific. His work on mechanics, fluids, optics, and acoustics makes it reasonable to call him a physicist *avant le lettre*. The Euler-Lagrange equations are crucial in theoretical physics to this day. Euler also discovered/invented the mathematics of *local* conservation laws, which are appropriate for continuous media (fluids and solids, and to some degree modern fields, as opposed to particles), namely the continuity equation, which involves partial derivatives with respect to time and space (Euler 1757). Euler took the conservation of energy (vis viva) to be not generically true on account of inelastic collisions (Euler 1746; Calinger 2016). Euler was a staunch interactionist dualist (Euler 1752, 1926) and a vigorous opponent of Leibniz–Wolff pre-established harmony (Euler 1840, I Letts. 79–115, II Letts. 1–17) and of monads (Broman 2012). He was an orthodox Christian and occasionally an apologist (Euler 1840, 1965; Arana 1994; Breidert 2007; Knobloch 2010; Drozdek 2010; Knobloch 2018). As such he can hardly have failed to notice that mental influence the physical violated conservation of momentum; evidently he just didn’t care. Euler could not have been the stout opponent of pre-established harmony and staunch interactionist that he was, as well as being the best physicist in the world, without having an informed opinion about the quite popular Leibniz–Wolff conservation argument. In short, the Leibnizian conservation objection, while holding the upper hand in Germany for a season, was opposed by great physicists implicitly and good philosophers explicitly during the eighteenth century and was driven back, with interactionism regaining the upper hand (Watkins 1995a, b, 1998; Priestly 1777, p. 64).

## Conservation of Vis Viva/Energy Revived

Various factors led to the revival of the conservation of vis viva/energy from the end of the 18th to the middle of the nineteenth century (Chang 2013; Smith 1998). Some empirical inputs included Count Rumford’s experiments on heat from boring of cannon and Sir Humphrey Davy’s melting ice by friction (Joule 1850). The fall of the caloric theory of heat opened the door for the mechanical equivalent of heat, putting empirical quantitative flesh on the old proposal that motion lost due to friction (such as from rubbing one’s hands together vigorously) is still motion, but now of insensibly small particles. Joule found the mechanical equivalent of heat using a paddle wheel experiment (Joule 1845, 1846, 1850; Young 2015). Helmholtz articulated a broad theoretical basis for conservation (von Helmholtz 1847). Thus the conservation of energy was revived apparently for good. There were many basically simultaneous partial discoverers including Mayer as well (Kuhn 1959; Cahan 2012). For the moment I omit what one might retrospectively call ‘high’ theoretical physics (the least action principle, the symmetry-conservation link, and local field theory), much of which was the province of mathematicians’ analytical mechanics in that era and was not considered generally applicable.

It was not long before the Leibnzian energy conservation objection to vitalism and to interactionist dualism (most obviously to soul-to-body causal influence) was revived during the 1860s–1880s by Helmholtz and Du Bois–Reymond (von Helmholtz 1861; Wegener 2009; van Strien 2015). Such authors argued for the reducibility of physiology to physics, which would exclude vital or mental forces. One type of response by vitalists and interactionist dualists involved almost-conservation. As Maxwell, Stewart, Cournot, and Saint-Venant pointed out in various ways, the delicate construction of life made possible mental or vital influence on the physical with only the tiniest non-conservation (van Strien 2015). By analogy to firing a gun, sailing a ship, or controlling a locomotive, a human decision can launch with control the bullet, ship or train without the mind’s supplying most of the energy to move the bullet, ship or locomotive; the mind simply makes a small exertion that directs the release of a large amount of physically or chemically stored potential energy. Thus interactionist dualism (or interactionism) is at least compatible with *the evidence for conservation*, which is never perfect, even if incompatible with strict conservation. An alternative view combined exact conservation and indeterminism (van Strien 2015). While the latter view has a certain elegance, it is harder to see whether it can work. Thus in the nineteenth century the conservation of energy resurfaced with a new name and on better grounds, now integrated into thermodynamics at the phenomenological level and gradually tied to symmetries and the principle of least action at the theoretical level.

## Leibniz’s Argument Against Interactionism Revived

Did the vindication of the conservation of energy also vindicate Leibniz’s argument against interactionism? At least sociologically it seemed to do so: the Leibnizian argument reasoning played a role in psychology from the nineteenth century onwards (Marshall 1982; Daston 1982; Heidelberger 2004; Wegener 2009). This was not inevitable on physical grounds: the implicit Newtonian view that momentum is not conserved due to mental causation (while vis viva is not conserved on more general physical grounds) could easily have been updated to a view that both momentum and energy are not conserved due to mental causation. While such a view has been taken by some, the argument from conservation laws against mind-body interaction has continued to inspire confidence within analytic philosophy to this day. According to Dan Dennett,

the conservation of energy...accounts for the physical impossibility of ‘perpetual motion machines,’ and the same principle is apparently violated by dualism. This confrontation between quite standard physics and dualism...is widely regarded as the inescapable and fatal flaw of dualism. (Dennett 1991, p. 35)

According to Mario Bunge,

*Dualism violates conservation of energy.* If immaterial mind could move matter, then it would create energy; and if matter were to act on immaterial mind, then energy would disappear. In either case energy—a property of all—and only concrete things would fail to be conserved. And so physics, chemistry, biology, and economics would collapse. Faced with a choice between these “hard” sciences and primitive superstition, we opt for the former. (Bunge 1980, p. 17)

Unfortunately Bunge’s catastrophe is short on argument; the solutions (exact or approximate) of partial differential equations in physics are typically fairly robust and tolerant of external sources (Jackson 1975) without yielding catastrophic instabilities. Another proponent of the Leibnizian energy conservation objection is Paul Churchland:

[non-epiphenomenalist] ...forms of Dualism do fly in the face of basic Physics itself, a rather more damning matter [than flying “in the face of the constituting convictions of Folk Psychology and the explanatory practices they sustain”], since any position that includes non-physical elements in the causal dynamics of the brain must violate both the law that energy is neither created nor destroyed, and the law that the total momentum in any closed system is always conserved. In short, you simply can’t get a change in any aspect of the physical brain (for that would causally require both energy changes and momentum changes) save by a compensatory change in some other physical aspect of the brain, which will thereby lay claim to being the cause at issue. There is simply no room in a physical system for ghosts of any kind to intervene in some fashion to change its dynamical behavior. Any physical system is ‘dynamically closed’ under the laws of Physics. (Indeed, it was this very difficulty, over a century ago, that motivated the desperate invention, by Thomas Huxley, of Epiphenomenalism in the first place.) (Churchland 2011)

In short, this sort of argument is widely accepted (Morowitz 1987; Pollock 1989, p. 19; Flanagan 1991, p. 21; Fodor 1998, p. 64; McGinn 1999, p. 92; van Inwagen 2009, p. 246; Searle 2004, p. 42) Lycan (2011) (Westphal 2016, pp. 41–44) (and more in lists in Montero 2006; Collins 2008; Gibb 2010; Pitts 2019). While many or most of those presenting this sort of argument are naturalists, presumably the acquiescence in the conservation of energy is intended as a submission to a scientific fact (a fact that provides confirmation for naturalistic philosophy) rather than as an assertion of naturalistic philosophy. A mere assertion of naturalistic philosophy would be dialectically inappropriate in an argument against interactionist dualism. A scientific fact, on the other hand, would fit the bill nicely.

While interactionist dualism has been a minority view in recent decades, a large part of this minority (or its sympathizers such as C. D. Broad) has responded to this objection by denying any incompatibility between such mental causation and conservation laws (Broad 1937; Gibb 2010; White 2017). Presumably these philosophers also take the acquiescence in the conservation of energy as a submission to a scientific fact rather than as a mere assertion of naturalistic philosophy. These philosophers often are not naturalists; while Broad was no theist, his acceptance of parapsychology/spiritualism (Broad 1937, 1953, 1962) at least involves the existence of spirits and their influence on the physical world. There would be little point in conceding that naturalism is true when one actually takes it to be false. But accepting scientific facts would be quite appropriate.

In an important respect Dennett, Bunge and Churchland are correct: the conservation laws and interactionist dualist mental causation are indeed basically incompatible.^4^ In this sense the anti-dualists are closer to the mark. The greater logical strength of local, as opposed to merely global, conservation laws^5^ leaves no room for hiding the soul’s influence by, e.g., compensation by the opposite amount of energy elsewhere.

Probably both of these groups are motivated by a view that the conservation of energy (and perhaps likewise the conservation of momentum) is a scientific fact of the form that energy can be neither created nor destroyed. While the modal force of “can” could be discussed, there is at least the implication that in the actual world, *energy is neither created nor destroyed*.

## Responses to Leibniz’s Argument

Ladyman et al. have warned us, however, that not everything aiming to be naturalistic metaphysics (in the sense of respecting science) is altogether successful.

We might thus say that whereas naturalistic metaphysics ought to be a branch of the philosophy of science, much metaphysics that pays lipservice to naturalism is really philosophy of A-level chemistry. (Ladyman et al. 2007, p. 24)

Is the conservation issue another example where A-level (advanced secondary school) chemistry is assumed in a context where the difference with graduate-level physics is significant?

Unfortunately the answer is “yes.” The former (anti-interactionist) view is a reasonably accurate grasp of what it would be like for the conservation laws to hold fully, but fails to recognize that the symmetry-conservation law link enshrined in Noether’s theorem (on which more below) imposes on them a burden to *argue that the conservation laws are true*—not just close approximations or true in most places and times (which no dualist should deny), but exactly true everywhere and always including brains. Consequently the argument assumes what was to be proven. This view also tends to overlook the locality of conservation laws and hence exaggerates the menace of non-conservation [e.g., (Bunge 1980, p. 17)]; conservation can hold perfectly well in astrophysics and refrigerators, even if interactionist dualism is true. The conservation-affirming interactionist view also tends to overlook the symmetry-conservation law link—why else would one work so hard to establish consistency when it seems so unpromising?—and often deploys a mistaken conception of conservation laws as primarily global (not local) in an effort to uphold conservation through nonlocal compensation. Nonlocal compensation amounts to trying to make up for a violation in one place with a violation in another place, repeating rather than mitigating the offense. The idea is unworkable in any case: it is absurd that either there be another soul(s) elsewhere requiring just the opposite amount of non-conservation (especially if souls are libertarian-free) or that energy conservation fail somewhere with no soul present.

It appears that only a minority of interactionist dualists in the last century or so has been willing to let the conservation laws fail, often with a partial awareness of the symmetry-conservation link (Ducasse 1960; Averill and Keating 1981; Larmer 1986; Plantinga 2007). As it happens, this sort of view (naturally with less grasp of the symmetry-conservation law link) was much more common in the eighteenth century (Watkins 1995a, 1998; Pitts 2020b). In the recent discussion, letting the conservation laws fail presumably has seemed like a bridge too far for all but the heartiest of *a priori* and/or religious metaphysicians, or the most informed about the symmetry-conservation relation (Averill and Keating 1981). Surprisingly enough, this view shows the best understanding of the relevant theoretical physics. Whether it shows a good view of *neuroscience* is of course a wholly separate question not addressed in this paper.

The failure of the argument from conservation laws against interactionism has been previously noted by Jeremy Butterfield (partly quoted as the epigraph):

This argument is flawed, for two reasons. The first reason is obvious: who knows how small, or in some other way hard to measure, these energy gains or losses in brains might be? Agreed, this reason is weak: clearly, the onus is on the interactionist to argue that they could be small, and indeed are likely to be small. But the second reason is more interesting, and returns us to the danger of assuming that physics is cumulative. Namely, the principle of the conservation of energy is not sacrosanct. The principle was formulated only in the mid-nineteenth century; and although no violations have been established hitherto, it has been seriously questioned on several occasions....And, furthermore, it is not obeyed by a current relevant proposal...for solving quantum theory’s measurement problem.

In short, physicalists need to be wary of bad reasons to think physicalism is true, arising from naivety about physics.

(Butterfield 1997, pp. 146, 147)

When we recall that Newton and Euler were both at least implicitly committed to accepting interactionism and letting the conservation laws fail, it is less surprising when those well versed in physics today also reject the Leibnizian argument.

## Conservation Laws Are Local

One of the key features of conservation laws in modern physics is that they are *local* (Lange 2002, chapter 5). The high school chemistry understanding of conservation laws, by contrast, apparently takes conservation to be simply $E=constant,$ a global conservation law. While there is talk about energy flowing from one place to another, there is rarely any suggestion that this flow is so disciplined as to be described by one (differential) equation at each point in space, as opposed to a single equation for the whole world. Expressed using single-variable calculus (a good step towards a more modern view), one can write the global conservation of energy $E=constant$ equivalently as $\frac{dE}{dt}=0.$ Electromagnetism and gravitation were subsumed into local field theory during the 1870s and the 1910s, respectively, removing the examples of action at a distance and making conservation in fundamental physics take much the same form that it had taken since the mid-eighteenth century for continuous media (fluids and solids). Now energy and momentum are understood to have locations and move at finite speeds.^6^

A local conservation law says in effect that for each little volume, the energy (or momentum or charge...) in the volume changes only insofar as energy (...) flows through its boundary. Thus there is no teleportation (whether instantaneous, at the speed of light, or at any other pace), no disappearance into nothing, and no appearance out of nothing. Mathematically one employs an energy density $\rho (t, {\mathbf{x}})$ and an energy current density ${\vec{\mathbf{J}}}(t,{\mathbf{x}}).$ A local conservation law takes the form $\frac{ \partial \rho (t,{\mathbf{x}}) }{\partial t} + {\vec{\nabla}} \cdot {\vec{\mathbf{J}}} (t, {\mathbf{x}}) = 0$ at each place and time: the rate of increase of the energy per unit volume at a given place and time, and the tendency of energy to spew out of that place and time (the “divergence” of the current density), together add up to 0. Thus if the energy per unit volume is going up at some time and place $\left( \frac{ \partial \rho (t,{\mathbf{x}}) }{\partial t} > 0 \right)$, then energy must be getting sucked in (negative divergence) rather than spewed out there and then: ${\vec{\nabla}} \cdot {\vec{\mathbf{J}}} (t, {\mathbf{x}}) < 0.$ The two terms must have opposite signs in order to add up to 0. This equation is called the continuity equation. Introducing the universal quantifier $\forall$ (for all) to make explicit that this equation is intended to apply at all places and times, one writes

$$\begin{aligned} (\forall t) (\forall {\mathbf{x}}) \left[ \frac{ \partial \rho (t,{\mathbf{x}}) }{\partial t} + {\vec{\nabla}} \cdot {\vec{\mathbf{J}}} (t, \mathbf{x})=0 \right] . \end{aligned}$$

This quantified equation is like a continuous conjunction of conservations in all the different places and times. The continuity equation, with detailed expressions for $\rho$ and ${\vec{J}}$ derived from the Lagrangian in question, is the conservation in the symmetry-conservation mutual implication of Noether’s first theorem.

If one uses the more expressively adequate component notation using $\nabla \cdot {\mathbf{J}} = \frac{\partial J_x }{\partial x} + \frac{\partial J_y }{\partial y} + \frac{\partial J_z }{\partial z}$, then one has

$$\begin{aligned} (\forall t) (\forall x) (\forall y)(\forall z) \left[ \frac{ \partial \rho }{\partial t} + \frac{\partial J_x }{\partial x} + \frac{\partial J_y }{\partial y} + \frac{\partial J_z }{\partial z} =0 \right] . \end{aligned}$$

Component notation generalizes more readily to General Relativity, in which one faces a choice between the continuity equation formally describing exact conservation of a partly mysterious combination (material energy + gravitational (pseudo-?) energy) and the elegant 4-dimensional vector/tensor character that lets one convert freely between components relative to coordinate basis and coordinate-independent bold-faced symbols, arrows with magnitude and direction, etc. (Anderson 1967). In component notation, one can express either the continuity equation (which employs a gravitational energy-momentum “pseudotensor,” which is difficult to interpret due to various real or alleged vices) or a tensorial equation that represents a balance law rather than a conservation law. Thus the component notation does not prejudge the content. In more elegant notation with bold-faced symbols, one can naturally express only the balance law, not the conservation law. One would need a good reason to abandon the conservation law, a reason which in my opinion at least does not exist.

## Gentle Failure of Conservation Laws

Writing out the logical form of a local conservation law in terms of quantifiers is useful because it helps to illustrate a form of robustness, in contrast to the catastrophe that Bunge fears. One knows that the negation of a conjunction is the disjunction of negations: $\lnot (A \& B) \leftrightarrow (\lnot A \vee \lnot B).$ Universal quantification is like a huge conjunction, while existential quantification is like a huge disjunction. Thus the negation of a universally quantified statement (in this case the continuity equation) tells us that conservation fails *somewhere at some time*, perhaps at many time-places. This claim can be made using the existential quantifier $\exists .$ Formally one has $\lnot \forall \leftrightarrow \exists \lnot ,$ so one has

$$\begin{aligned} \lnot (\forall t) (\forall x) (\forall y)(\forall z) \left[ \frac{ \partial \rho }{\partial t} +   {\vec{\nabla}} \cdot {\vec{\mathbf{J}}} =0 \right] \leftrightarrow (\exists t) (\exists x) (\exists y)(\exists z) \left[ \frac{ \partial \rho }{\partial t} +  {\vec{\nabla}} \cdot {\vec{\mathbf{J}}} \ne 0 \right] . \end{aligned}$$

Here the negation of an equality has been rendered as an inequality, which seems defensible if interpreted sympathetically; it is possible, however, that the mathematical expressions simply fail to make sense and hence fail to justify an inequality. Hence the interactionist wants to say that conservation holds in most times and places, but fails in a few, namely, where souls act on brains. This claim is easier to express in ordinary English than in formal logic.

If conservation fails somewhere sometime, that is not nearly as frightening as if it failed everywhere all the time. Note that without the explicit quantifiers, one might think that the failure of the conservation law would be $\frac{ \partial \rho }{\partial t} + \frac{\partial J_x }{\partial x} + \frac{\partial J_y }{\partial y} + \frac{\partial J_z }{\partial z} \ne 0,$ and one might think that $\frac{ \partial \rho }{\partial t} + \frac{\partial J_x }{\partial x} + \frac{\partial J_y }{\partial y} + \frac{\partial J_z }{\partial z} \ne 0$ means nonconservation everywhere and always. (Perhaps that is the catastrophe that Bunge feared.) Fortunately such a conclusion would be logically fallacious, as is clear once the quantifiers are made explicit. Non-conservation in brains, though surprising, would not threaten the thermodynamics of refrigerators or astrophysics. While the use of quantifiers might suggest the academic level of a university-level formal logic course, that is not correct. One can easily understand, for example, that if a box isn’t brown and large, then it might be brown and not large, or large and not brown, or neither brown nor large. If something isn’t true everywhere and always, then it is false (at least) somewhere sometime. Much as there was no need for introducing the continuity equation and its concomitant mathematics of multi-variable differential calculus for expressing the basic idea of the continuity equation, there is no need for introducing quantificational logic to point out that the failure of conservation might be a quite isolated affair rather than a global catastrophe. Clearly such ideas are within reach at the secondary school level.

Note also that if conservation laws fail somewhere sometime, it might be the case that this failure is very small: only a little bit of energy comes into being or disappears on account, e.g., of the soul’s influence. Newton’s laws of reasoning, we recall from above, allowed for mathematical relations that are only approximate. Various nineteenth century authors entertained the possibility that a slight deviation from conservation might suffice for mind-to-body causation (van Strien 2015). If nonconservation is sufficiently small in magnitude, or occurs only in places where one does not look, it might go undetected. This is yet another way that Bunge’s catastrophe could fail to occur.

## Symmetries Imply Conservation Laws and Vice Versa

Philosophers and others have discussed the idea of the natural world’s being unaffected by anything else under a few different terms. In the nineteenth century the “uniformity of nature” was a popular topic in relation to the foundations of geology and in relation to inductive inference. There is also talk about open versus closed systems, especially in relation to closed systems. Nowadays physicists and philosophers of physics talk about “symmetries” (Brading and Castellani 2003), especially but not only symmetries of the Lagrangian density, a mathematical function of the physical fields and their rates of change. In classical field theory, the fields’ equations of motion can be derived from the Lagrangian density. In the path integral approach to quantum field theory, the Lagrangian density is again the starting point. The uniformity of nature (in time and place) picks out some of the key symmetries that might hold for a Lagrangian density. In that respect, at least as long as a Lagrangian density is the right place to start physical theorizing, the benefits of talking about uniformities of nature are retained by talking about symmetries. Nineteenth century results about the relationship between symmetries and conservation laws (Lagrange 1811; Hamilton 1834; Jacobi 1996) were generalized and synthesized by Max Born and then Emmy Noether during the 1910s (Born 1914; Noether 1918). Born noted that for theories of continua (field theories), conservation of energy and of momentum hold when the Lagrangian density is independent of time and of place, respectively. Noether proved a more general result that includes Born’s result, along with a converse: conservation laws imply symmetries. Contrapositively, one could say that non-symmetry implies non-conservation. The symmetry-conservation link runs in both directions: symmetry $\leftrightarrow$ conservation law (Noether 1918; Brading 2001; Kosmann-Schwarzbach 2011).

A disadvantage of talk about open versus closed systems is that it is not obvious how these terms apply when one is considering the possibility of immaterial influences. If I have a soul that acts on my brain, is the physical world still a closed system? While the interactionist might say “no,” the answer is somewhat less clear given the fairly common view [albeit contested both a few centuries back and recently (Grant 1981; Pasnau 2011; Pitts 2019)] that spirits are not spatially located. If there is no soul in my brain, isn’t that enough for applying conservation to my brain, saying that the amount of energy in my brain or any part thereof is conserved except insofar as energy flows through the boundaries? Occasionally one sees energy conservation invoked as an objection to miracles as well (Stoeger 1995; Fales 2010, p. 13), though probably most people figure that a being said to create the world ex nihilo is perfectly free to effect energy non-conservation.^7^ Few have shared Newton’s view that God’s omnipresence implies being *located everywhere*, so the absence of spirits from space is likely a poor criterion for a closed system at least in the divine case. Fortunately, *questions about the location of spirits, finite or infinite, are entirely irrelevant, if one talks about symmetries of the Lagrangian density, or even about uniformities of nature, rather than open/closed systems*. Symmetry/uniformity talk puts the emphasis on where spirits *act*, not on where they are—which is highly desirable because the location of spirits has been a difficult question from the Scholastics onward. Thus it is clear that spirits acting on matter falsify symmetries/uniformities of nature in those space-time regions where the spirits in question act (if they exist and act on the physical world, that is). Hence spiritually speaking, spirit-to-matter causation implies an open rather than a closed system, but talking about symmetries or uniformities instead is greatly illuminating.

Given the symmetry-conservation law link, what, if anything, follows for the Leibnizian objection? If I have a soul that acts on my brain, it does so on Earth during my life, not on Mars 100 years ago. That soulish action on the physical world is akin to an externally applied potential, not to a physical entity with its own physical dynamics described by its own terms in the Lagrangian density. One is familiar with external potentials in modelling gravity as providing potential energy *mgz* for a point mass at height *z* above the Earth (assuming *z* is sufficiently small); that the point mass might generate gravity of its own or set in motion the Earth and hence alter the Earth’s gravitational field is not denied, but it is neglected mathematically. If gravity is a real physical entity (which it is), then such a treatment is only a frequently useful approximation in our circumstances, not a reflection of how gravity really is. On the other hand, the soul surely has no physical dynamics. While its workings are presumably somehow sensitive to the state of the physical world, and the soul presumably somehow acts on the physical world (at least according to the view under critique), the dynamics of the soul should involve beliefs and desires, a space of reasons, not pushes and pulls, or pressures, energy densities and momentum fluxes or other physical causes. I smell a hot pizza (matter-to-spirit influence). I decide to walk toward the pizza, pick up a piece, and eat it (a typical instance of belief-desire folk psychology with spirit-to-matter interaction). The taste motivates me to eat some more pizza. Given interactionist dualism (which must be assumed provisionally in order to generate a refutation from energy conservation), the physical world’s evolution is punctuated by influence from an immaterial realm not describable by Lagrangian field theory, though the influence itself presumably is so describable. Thus the physical equations of motion involve the mind’s influence as an external potential depending on time and place, analogy to a particle subject to gravity or a particle in an electromagnetic field (textbook physics problems). Consequently energy conservation and momentum conservation will not be true where and when the soul is acting.

Much as a particle in an applied gravitational field (that is, one in which one neglects the sources and dynamics) can fail to conserve momentum, one can have time-dependent Lagrangians in a biological context.^8^ Now two issues arise: neglecting for the dynamics of the environment (which causes the dependence on time) and distinguishing between fundamental *versus* higher-level descriptions. Clearly there is nothing surprising about a time-dependent Lagrangian due to the dynamics of the environment; one might simply be uninterested in anything more than its influence on the system in question. Then the system’s energy is not conserved, but presumably the dynamics of the system plus environment, should one bother to consider it, would conserve energy (and momentum). But the disanalogy to mind-body interaction is evident: whether one considers the dynamics of the brain or the dynamics of the whole physical world, one still has a time-dependent influence due to the influence of the non-physical mind, which has no dynamical equations from a Lagrangian. This point remains whether one envisages a description with the mind acting on fundamental physical fields (electrons, electromagnetism, and perhaps quarks, say) or a macroscopic description with the mind acting on higher level entities (parts of the brain). Clearly the latter description would be of more relevance to neuroscience, however.

## Circularity of Conservation Objection

The trouble for the Leibnizian objection is the need to show why the interactionist dualist should apply *modus tollens* rather than *modus ponens*. The Leibnizian claims that there is some absurdity or refutation here. What is it? Why should the interactionist not simply accept non-conservation as a consequence? Often one sees proponents of the Leibnizian objection simply introducing the conservation of energy as a premise. Isn’t the conservation of energy a Fact, one known since the nineteenth century and taught in secondary school? If energy conservation is a Fact, then the Leibnizian argument works. Like Leibniz, physicalist philosophers of mind typically believe that physics *categorically* shows energy conservation. But in light of the discussion of local conservation laws and the symmetry-conservation law relationship, the conservation of energy *in the times and places in question* is not a fact, but rather a tendentious assertion that has little or no claim on the interactionist—little or no claim, that is, except insofar as empirical study of the brain has licensed it. The fact that energy is conserved in steam engines and refrigerators is highly relevant evidence assuming that one formerly believed that souls act on steam engines and refrigerators. But no one has ever thought that. Thus introducing the conservation of energy as a premise simply begs the question against interactionist dualism.

As Aristotle pointed out in the *Posterior Analytics*, premises in a scientific demonstration must be better known than and prior to the conclusion (Smith 2015). Similar criteria are appropriate for making an argument that ought to change someone’s mind. But given Noether’s theorem and converse, symmetries and conservation laws are equally well known, and neither is prior. The claim that souls act on bodies clearly violates symmetries: my decision to eat pizza now affects my brain now, not the Sun years ago. With symmetries failing where and when my soul acts on my brain, the conservation of energy and momentum fail in my brain.

How should an interactionist respond? A common bad reply is denial that conservation is violated (Broad 1937; Gibb 2010; White 2017). This denial is presumably motivated by the belief that conservation laws are categorical, so that the Leibnizian objection would be potent if conservation failed. But given local conservation laws and the two-way relation between symmetries and conservation laws, conservation indeed does fail. So this response is not available. But it is also by no means clear why the physically well-informed interactionist should want to avoid this conclusion.

A better answer is, “yeah, nonconservation, so what?” A sufficiently small non-conservation that happens sufficiently rarely and in places that have not been carefully examined is not absurd on *physical* grounds. One should try to do the philosophy of real physics, not the philosophy of A-level chemistry, to generalize an old warning (Ladyman et al. 2007, p. 24). The conditionality of conservation has been noted occasionally in the last century or so (Ducasse 1960; Averill and Keating 1981; Larmer 1986; Lowe 1992; Plantinga 2007), but little heeded. What has been missing, presumably, is a sufficiently detailed treatment to show that this view is not a reactionary claim by religious or *a priori* metaphysicians insufficiently acquainted with science (as many seem to have believed—recall the Dennett, Bunge and Churchland quotations above), but rather a clear consequence modern physics (apart from General Relativity) (Pitts 2019; Cucu and Pitts 2019; Pitts 2020a). The conditionality of conservation was also the view of many of the best minds in centuries past, including Descartes (likely), Newton (likely), Knutzen, Crusius, Euler (likely), and Maxwell (sometimes) (Garber 1983; Watkins 1995a, 1998; van Strien 2015; Pitts 2020b). On closer investigation, Dennett’s “initial allegiance ...to the physical sciences” (Dennett 1994) does not actually pay off in diagnosing an “inescapable and fatal flaw” of dualism using quite standard physics.

Rejecting the Leibnizian conservation objection does not, of course, imply that one takes interactionist dualism to be true or even plausible. What does follow (apart from General Relativity) is that any decent argument will be empirical and will involve neuroscience (Thompson 2008). If there exist scientists who have the evidence against interactionism, those experts are neuroscientists, not physicists.

## What Difference Does General Relativity Make?

Thus far I have paid little attention to an elephant in the room, namely, the 100+-year controversy over the status of energy and conservation in General Relativity. One can find well-informed people who deny that there are conservation laws in General Relativity. One can also find people who say that while distorting Noether’s (first) theorem. I will start with some mathematical facts that, in my opinion, are some of the most important things to say about the subject.

When Max Born noted that the absence of *t*, *x*, *y*, or *z* from the Lagrangian density of a local field theory implies a conservation law (Born 1914), he made a statement of even broader applicability than he perhaps intended given his context of special relativistic field theories. His statement is true even for non-relativistic field theories and for General Relativity as well (Bergmann 1958; Goldstein 1980, p. 555). (Einstein seemed not to notice this, because he worked hard to show conservation by some other means and wrote that “Grossmann and I believed that the conservation laws were not satisfied” (Pitts 2016).) Whereas the rigid translations yielding conservation in Special Relativity are described by a finite number of parameters, in General Relativity one has infinitely many due to four arbitrary functions worth of coordinate freedom. In effect one can apply Noether’s first theorem in infinitely many different ways, getting results bearing no simple relationship. (One person’s definition of sitting still and waiting (time translation) is someone else’s definition of wiggling around a bit.) Traditionally this fact has been interpreted as counting against the objective localization or gravitational energy (Misner et al. 1973, p. 467) or perhaps even against its reality due to having incompatible properties (Hoefer 2000; Duerr 2019); another possible interpretation is that the different symmetries pick out different energies (Pitts 2010). Noether (1918) also showed that the total energy-momentum in General Relativity, unlike earlier theories, consists of a term proportional to the field equations for gravity only and a “curl” term with automatically vanishing divergence. More familiar theories would have an additional nonzero term giving the value of the conserved quantities and would have terms relating to the field equations for all of the fields, not just the gravitational field. Is conservation missing or trivial in General Relativity? Some have concluded so.

Given the Noether-based connection between symmetries and conservation laws, it seems to me an odd interpretive move to say that a theory with *more* symmetries of the Lagrangian density has *fewer* conservation laws, or even no or only trivial conservation laws. Noether’s (first) theorem yields infinitely many conserved currents. What are the usual objections to these quantities? One of the two main objections is that the gravitational energy term is pseudotensorial, not tensorial: the gravitational energy at a point depends in a radical way on the coordinate system, with no translation scheme (transformation rule) relating pseudotensor values in different coordinate systems. A common interpretation is that formal gravitational energy is generated or destroyed simply by a changed labelling of space-time, so such expressions should not be interpreted realistically. Instead gravitational energy is “not localizable.” A second objection is that there is a radical non-uniqueness of the gravitational pseudotensor, some standard examples belonging to Einstein, Papapetrou, Landau-Lifshitz, etc.

If one finds these objections impressive (and many or most people have), then one still faces the mathematical fact that there exist formal conserved currents arising from symmetries of the *Lagrangian density* (not the geometry) involving (formally) rigid translations in accord with Noether’s first theorem, much as in earlier theories (Schmutzer 1972). The equations do not disappear or become false by virtue of failing to meet some interpretive criteria. These currents take the form $\sqrt{-g}T^{\mu }_{\nu } + \sqrt{-g}t^{\mu }_{\nu }$ involving material energy-momentum $\sqrt{-g}T^{\mu }_{\nu }$ and gravitational (pseudo-?) energy-momentum $\sqrt{-g}t^{\mu }_{\nu }$. The sum $\sqrt{-g}T^{\mu }_{\nu } + \sqrt{-g}t^{\mu }_{\nu }$ satisfies the continuity equation (everywhere and always)

$$\begin{aligned} \sum _{\mu =0}^3 \partial _{\mu } \left(\sqrt{-g}T^{\mu }_{\nu } + \sqrt{-g}t^{\mu }_{\nu }\right) =0. \end{aligned}$$

In these respects gravitational (pseudo-) energy is just like the energy for any other field. The material energy-momentum itself satisfies only

$$\begin{aligned} \sum _{\mu =0}^3 \nabla _{\mu } \left(\sqrt{-g}T^{\mu }_{\nu }\right) = \sum _{\mu =0}^3 \partial _{\mu } \left( \sqrt{-g}T^{\mu }_{\nu }\right) - \sum _{\mu =0}^3 \sum _{\alpha =0}^3 \sqrt{-g}T^{\mu }_{\alpha } \Gamma ^{\alpha }_{\mu \nu }=0, \end{aligned}$$

where the Christoffel symbols $\Gamma ^{\alpha }_{\mu \nu }$ depend on the metric tensor and its first derivatives. This latter equation generally cannot be integrated to yield constant energy and constant momentum (even with favorable boundary conditions) due to the second term (Weyl 1922, pp. 236, 269–271; Misner et al. 1973, p. 465; Lord 1976, p. 139). Hence the sum of material energy + gravitational (pseudo-?) energy is locally conserved and (if boundary conditions permit) globally conserved, while material energy usually is not locally or globally conserved. Clearly the gravitational (pseudo-?) energy expression $\sqrt{-g}t^{\mu }_{\nu }$ looks, walks and quacks in many respects like energy; are these respects sufficient to interpret it as energy? (Recently the reality of gravitational energy in at least some contexts has been defended on functionalist grounds (Read 2020).) The typical physics textbook view is that one should not take the local conservation involving $\sqrt{-g}T^{\mu }_{\nu } + \sqrt{-g}t^{\mu }_{\nu }$ seriously, but one should take seriously the spatially integrated conservation law when boundary conditions permit, thus also picking out a small collection of contingently physically preferred coordinates (Misner et al. 1973). This orthodoxy has seen increased resistance in recent decades, however. How does gravitational energy manage to be objectively nowhere in particular while having a superabundance of local descriptions and also good global behavior?

A common confusion is to consider symmetries of the *space-time geometry* as required for conservation laws. For theories prior to General Relativity, this requirement is reasonable. But Noether’s theorem, which applies even to General Relativity, looks for symmetries of the Lagrangian density, not symmetries of the geometry (Noether 1918; Trautman 1966). The point is that the space-time metric in General Relativity has its own field equations and hence is relevantly similar to matter; thus the metric does not need to have symmetries in order for Noether’s theorem to find symmetries and conservation laws.

Confusion on this point (a complaint that the metric lacks “motions,” that is, Killing vector fields, symmetries) lay at or near the root of a high-profile gravitational heresy led by Soviet/Russian Academician A. A. Logunov for decades (Logunov and Folomeshkin 1977; Denisov and Logunov 1982, critiqued Faddeev 1982; Zel’dovich and Grishchuk 1986, 1988). Robert Gentry’s complaints about nonconservation as an absurdity of Big Bang cosmology are analogous (Gentry 1998; Gentry and Gentry 1998) (critiqued Pitts 2004a, b).

Other authors similarly think that symmetries of the geometry (as opposed to the Lagrangian density) are needed for conservation laws, but interpret the inferred lack of conservation laws as a *feature* of General Relativity, or at least as an acceptable consequence, rather than a bug (Motl 2010; Hossenfelder 2016; Physics Stack Exchange 2017; Hossenfelder 2018; Siegel 2018; Maudlin et al. 2020). But Noether’s theorem has no concept or role for geometry or symmetries thereof: there are only fields in the Lagrangian density and symmetries of the Lagrangian density. Thus General Relativity really does have Noether-based conservation laws, albeit with unfamiliar and in some respects unattractive qualities. Carroll, while preferring the nonconservation view and showing no clarity about Noether’s theorem, regards the topic as a matter of interpretive choice (Carroll 2010).

The idea that General Relativity lacks conservation laws might lead one to expect various consequences that are in fact very questionable. Besides above-cited claims that this lack yields a refutation of the theory, one also finds authors who *exploit* the supposed nonconservation as a resource for energy non-conserving processes. Russell Humphreys claimed that this non-conservation was a resource for creation science to dispose of heat from accelerated nuclear decay (Humphreys 2000) (critiqued Pitts 2009b).

More relevant to present purposes is the invocation of this claimed non-conservation to respond to Leibniz’s conservation objection to interactionist dualism (Mohrhoff 1997; Collins 2008, 2011). Unfortunately this claim does not work: for pre-General Relativistic theories this answer is unnecessary (the symmetry-conservation link sufficing to show the question-begging character of Leibniz’s objection), but for General Relativity it is false (Pitts 2020a). As one can demonstrate using the Bianchi identities, thus avoiding questions of interpreting pseudotensors, General Relativity has some tendency (though not all that much) to resist the introduction of external influences, unlike earlier theories. One can show that Einstein’s equations force up to four scalar fields worth of would-be mental causation to vanish; at that point the resistance is broken. If one believes that General Relativity conserves energy, one *can* interpret this claim in terms of a strengthened neo-Leibnzian energy conservation objection, one that doesn’t so obviously beg the question. Those who interpret General Relativity as not conserving energy can still use the Bianchi identities to draw this conclusion. The fact that the non-conservation interpretation’s heuristic force is directly opposed to the actual mathematics should be noted, however.

The two traditional objections to pseudotensors are less compelling now than they were over 20 years ago. According to James Nester and collaborators, the non-uniqueness problem is a feature rather than a bug: the different pseudotensors all have physical meaning in relation to different boundary conditions (Chang et al. 1999, 2000; Nester 2004). One can interpret the coordinate-dependence of pseudotensors as the kind of property needed in order to represent the infinity of conserved energies (Pitts 2009a, 2010) that must exist to correspond to the infinity (Bergmann 1958) of symmetries of the Lagrangian density. If a 10- or 16-component expression for gravitational energy had a transformation rule to show the equivalence of the values in different coordinate systems, then only one energy would be expressed, not infinitely many as is required. Coordinate systems are somewhat analogous to natural languages; tensor calculus is akin to publishing every book translated into every language, whereas a pseudotensor as akin to publishing Shakespeare in English, Goethe in German, *etc.*, using far less paper to express the same ideas to a sufficiently capable reader. These two infinities (from nonuniqueness and coordinate dependence) might turn out to be the same infinity:

...the totality of all conservation laws ${\bar{C}}^{\rho },_{\rho }=0$ in one coordinate system is equivalent to one of them, stated in terms of all conceivable coordinate systems. (Bergmann 1958)

In light of the infinity of rigid symmetries, one infinity is desirable. Hence the door is less closed than it once seemed regarding taking the Noether pseudotensor mathematics seriously.

What can one say about energy conservation and General Relativity to a secondary school audience? Clearly the locality of energy conservation still holds mathematically, though there are questions of interpretation. Whereas pre-General Relativistic theories passively accept non-uniform external influences due to the if-and-only-if relation between symmetries and conservation laws, General Relativity has some tendency to exclude such influences, although not a very strong one.

## How to Improve Secondary Education About Conservation Laws

The views that most non-academics and probably most philosophers have about conservation laws seem to be largely based on secondary school chemistry. The mathematics involved in providing a proper statement of conservation laws, which are local, involves multi-variable differential calculus to describe how quantities vary with time while leaving place alone, or vary with location in the *x*-direction at constant time and constant *y*- and *z*-coordinates. Such mathematics tends to be learned (at least in the American system, which is familiar to me) in the second year of an undergraduate education in engineering, mathematics, or physical science, but likely is not learned at all if one specializes in another subject. The most basic features of the symmetry-conservation law relation tend to be learned in the third year of an American undergraduate education in physics using a mechanics book like (Marion and Thornton 1988). Most of the machinery appears in graduate-level physics (Goldstein 1980, chapter 12), if one encounters it in coursework at all. Such material is not and should not be part of a standard philosopher’s education. Hence neither locality nor (bi)conditionality of symmetries is likely to be widely known among philosophers [but see (Lange 2002, chapter 5)]. It therefore isn’t necessarily anyone’s fault that philosophers build arguments around an inadequate understanding of conservation laws. It is natural to assume that one’s secondary school science education would be refined and extended, and perhaps subtly amended, but not fundamentally contradicted by university-level or even graduate-level study.

Unfortunately the line between subtle amendment and fundamental contradiction is unsustainable given the exacting uses that some philosophers aim to make of conservation laws. Is the following claim a subtle amendment to or a fundamental contradiction of the lesson from secondary school chemistry? “Conservation holds exactly almost everywhere and always, but suffers small failures at times in the brains of living persons and perhaps higher animals.” One might think that the quoted claim is a subtle amendment of the secondary school chemistry story. But the quoted claim is useless for a Leibnizian argument. The Leibnizian argument makes use of conservation in a way that is not robust or stable in something like the physical sense; under small perturbations, the argument goes away. Is the claim in quotation marks *true*? That is primarily an empirical question—one answered not by looking at steam engines or laboratory flasks or thigh muscles, but at the *brains* of the beings in question.

While Lagrangian local field theory and the continuity equation are likely to remain outside the education of most philosophers and are certainly out of reach for secondary school chemistry classes, ordinary language approximate paraphrases are certainly possible. Here is an approximate paraphrase of the locality of conservation:

Energy is located in particular places, and conservation is primordially local. Energy does not disappear in one place and reappear in another, or simply disappear, or simply appear; it only moves around. The amount of energy in some region changes only to the degree that energy flows into or out of the boundaries of the region. In some contexts, it is possible to add up all the conservation laws describing how energy moves around and infer that the total amount of energy in the universe remains constant. But cosmology suggests that one cannot do that in the real world.

This statement clearly does not require university-level mathematics in the form of multi-variable differential calculus to state or understand. An approximate paraphrase of the symmetry-conservation law link, neglecting locality at this stage, might go like this:

Energy is conserved if and only if the laws are uniform over time. Momentum is conserved if any only if the laws are uniform across space.

Putting locality and the symmetry-conservation law link together, one might say this:

Energy is conserved at a given time and place if and only if the laws there and then do not vary with time. Momentum is conserved at a given time and place if and only if the laws there and then do not vary with place.

These approximate paraphrases admittedly do not carry the full content of the exact statement (at least neglecting quantum physics) (Cucu and Pitts 2019; Goldstein 1980, p. 555):

$$\begin{aligned} (\forall x^0)(\forall x^1)(\forall x^2)(\forall x^3) \left( \sum _{\nu =0}^3 \frac{\partial }{\partial x^{\nu } } T^{\nu }_{\mu } = - \frac{\partial \boldsymbol{\mathcal {L}}}{\partial x^{\mu }} \right) . \end{aligned}$$

Here $T^{\nu }_{\mu }$ applies to all physical fields together; gravity is not singled out for special treatment. This quantitative statement also implies, for example, that a weak explicit dependence of the Lagrangian density on time implies a little energy non-conservation, whereas a strong explicit dependence of the Lagrangian density on time implies a lot of energy non-conservation. But the approximate paraphrases do carry far more content than is generally conveyed in secondary school chemistry and enough to understand the failure of Leibniz’s objection due to begging the question. The equation also shows that symmetries and conservation laws can be patchy, applying in most but not all of the world.

The difference made by General Relativity has been discussed above.

## Why Does It Matter?

There are a number of reasons, both truth-related and action-related, for reforming secondary school teaching about the conservation of energy to become more accurate. First, for the sake of truth, one should not confuse a philosophical thesis about the absence of spirit-to-matter influence (perhaps supportable by arguments from a Spinoza or a Hume) with a result of the physics of matter (perhaps supported by work by a Joule or a Mayer). Some of the discoverers of energy conservation would agree.

Second, such confusion is likely to engender in some circles a wider suspicion of science as embodying a naturalistic agenda. If ‘science denial’ is presently considered problematic, then making shoddy arguments in the name of science is not the way forward; rather such arguments provide *good reasons* for denying claims marketed as scientific. The Leibnizian objection, though marketed as scientific, is indefensible, so making or facilitating this argument diminishes the credibility of science. The interesting scientific evidence in the neighborhood comes from neuroscience, not thermodynamics. General Relativity makes a difference, but this difference is on the cutting edge of research and hence perhaps in need of seasoning before popularization.

Third, intensified suspicion of claims marketed as scientific will also retard significant action taken regarding climate change. To effect such change, especially in the United States—a place where there exists an unusually strong combination of popular resistance to scientific authority and large-scale combustion of fossil fuels—science and science teaching must be seen as fact-based rather than as philosophically tendentious. This paper has discussed an opportunity for further improvement.

Fourth, regarding the history of science, reforming teaching on conservation laws makes it easier to understand what some nineteenth century proponents of conservation laws (who in some cases made exceptions for Creative Power, for example) were saying and were not saying. Thus one can see the highly non-trivial innovation proposed by Helmholtz in 1861 that was not in his earlier work (von Helmholtz 1847, 1861).

## Footnotes

1 One might think that God’s creating the world with this harmony includes a case of mental-to-physical causation if God is a mind or is relevantly like one and yet creates matter. Leibniz seemed not to find God’s creating the world objectionable (Alexander 1956, pp. 75, 76).

2 That is, act non-redundantly. In contrast to the eighteenth century debate (to my knowledge), the current debate entertains the conceptual possibility of mental causation that makes something happen that physical causation would have made to happen anyway. Thus nowadays it is possible to affirm mental causation without the mind’s making any real difference. This conceptual possibility is often rejected by denying that there is systematic overdetermination.

3 One might think that a Newtonian could appeal to Newton’s laws for reasoning in support of exact and exceptionless conservation laws. (I thank Shaul Katzir for this suggestion.) Such a move might be even more appealing today than it was for historical Newtonians. The third and especially fourth rules of reasoning are relevant. The fourth rule says: In experimental philosophy, propositions gathered from phenomena by induction should be considered either exactly or very nearly true notwithstanding any contrary hypotheses, until yet other phenomena make such propositions either more exact or liable to exceptions. This rule should be followed so that arguments based on induction may not be nullified by hypotheses. (Newton 1999, p. 442) I suggest that Newton’s fourth rule gives two different potential escape routes for the interactionist. One possible loophole for the interactionist is to say that the non-conservation is small and hence compatible with conservation’s being “very nearly true” even if incompatible with its being true (van Strien 2015; Butterfield 1997, pp. 146, 147). Another, perhaps more promising loophole is to argue that other phenomena making conservation liable to exceptions are known or highly plausible, namely, mental causation. Newton’s own above-cited views on mental causation (Dempsey 2006; McGuire 1968, pp. 169, 170) indicate that he might well have agreed with the invocation of the second loophole.

4 Subtle forms of reconciliation might avoid a contradiction while giving up much of what one expect an interactionist position to involve (Lowe 2003).

5 This statement assumes for simplicity that the local laws can be integrated to give a global law, a process that can fail to make sense, for example, if there is too much activity no matter how far out one goes (Peebles 1993, p. 139). Without that qualification, local conservation is not logically stronger than global conservation.

6 Actually conservation laws in General Relativity has been controversial since the 1910s. One can safely say that mathematical equations of the usual conservation form arise in General Relativity due to symmetries of the laws, as in other theories. Whether one can interpret the results realistically is a more difficult question. Fortunately, that question does not need resolution here, though it will be discussed further below.

7 Amusingly, some calculations of the energy of the universe in General Relativity give 0, so it isn’t obvious that creation ex nihilo, or for that matter evolution ex nihilo, conflicts with global conservation (Tryon 1973; Rosen 1994; Johri et al. 1995; Banerjee and Sen 1997; Thirring 2003). Local conservation is a more difficult question.

8 I thank a referee for mentioning this example.
